# Micromechanical Observation and Numerical Simulation for Local Deformation Evolution of Duplex Stainless Steel

**DOI:** 10.3390/ma15228076

**Published:** 2022-11-15

**Authors:** Jian Zhao, Yanru Shi, Sujuan Guo, Mingliang Zhu

**Affiliations:** 1 School of Aerospace Engineering and Applied Mechanics, Tongji University, 1239 Siping Road, Shanghai 200092, China; 2 Key Laboratory of Pressure Systems and Safety, Ministry of Education, School of Mechanical and Power Engineering, East China University of Science and Technology, Meilong Road 130, Shanghai 200237, China

**Keywords:** duplex stainless steel, nanoindentation, digital image method, finite element, local stress and strain distribution

## Abstract

The characteristics of local strain distribution and evolution of duplex stainless steel during the tensile process were studied using the digital image correlation (DIC) technique. In addition, the finite element inversion of nanoindentation experiments of austenitic and ferrite phases in duplex stainless steel was carried out to obtain the stress–strain response of the two phases. Further, based on the representative volume element (RVE) and the material parameters obtained from the finite element inversion method, the local stress and strain behavior of duplex stainless steel at microscale was simulated numerically. The results fit well with the experiments, showing that the austenite phase is softer than ferrite phase, with the larger strain zone concentrated in the austenite phase and the larger stress zone concentrated in the ferrite phase. The grain boundaries are prone to obvious stress and strain concentrations. The local stress and strain distributions are influenced by the shape and interaction of the grains, while the distribution features become more obvious as the load increases. The research results effectively reveal the two-phase interaction and local failure mechanism of duplex stainless steel, and may provide a reference for material preparation and safety design of related structures.

## 1. Introduction

The microstructure of duplex stainless steel contains almost equal proportions of ferrite and austenite, which combines the excellent properties of ferritic steel and austenitic steel. Duplex stainless steel has been widely used in industry applications [1,2,3,4,5,6,7] due to its good plasticity and toughness combined with high strength, corrosion resistance, and good weldability [1,2,3,4,5,6,7]. Its mechanical behavior can be explained from the microstructure, which is predominantly composed of a soft austenite matrix with hard ferrite particles. Hard ferrite provides substantial strength, while the soft austenite phase is associated with good ductility.

Due to the discontinuous mechanical properties of duplex stainless steels, significant stress concentrations can easily occur even under small macro loads [8], which may lead to large local plastic deformation and failure. Therefore, it is crucial to investigate the local stress–strain characteristics of each phase material of duplex stainless steel. Common experimental measurements of the macroscale or local stress and strain behavior include digital image correlation (DIC), electron backscatter diffraction (EBSD), nanoindentation, and more [9,10,11,12,13]. In recent decades, many researchers have systematically studied the local deformation behavior of duplex stainless steel [14,15,16,17,18]. Metro [14] used image analysis techniques to observe the local deformation behavior of individual grains. After in situ tensile tests on rod-shaped and plate-shaped duplex stainless steel, it was found that the changes of local microstructure are closely related to the shape of each phase of the materials. Fréchard [15] combined atomic force microscopy (AFM) and EBSD methods to perform in situ tensile tests on duplex stainless steel, which showed that stress concentrations occurred at the grain boundaries. In addition, Bartali [16] calculated the displacement and strain fields of duplex stainless steel at the microstructural scale from surface images during cyclic loadings using the DIC technique, showing strain inhomogeneities and possible crack initiation position at the microscale. Liu [12] conducted a tensile test on 2205 duplex stainless steel at 250 °C and observed it using DIC technology, finding that the uneven strain field mainly appeared in austenite and occasionally appeared at the phase boundary. The above studies used a variety of test methods to show the local microdeformation and regularity of duplex stainless steel. However, they were not able to analyze the behavior and evolution of local stress and strain in the material, nor to explain the interaction mechanism between the two phases and grain boundaries. Nanoindentation experiments can be used to further investigate the deformation behavior of duplex stainless steels. Cui et al. [17] analyzed and compared the mechanical properties of the austenite phase and ferrite phase, finding that austenite is softer than ferrite and that phase transformation did not occur during the nanoindentation process. However, the tensile curves of the two phases were not inverted, and the stress–strain relationship of the two phases could not be characterized. Furthermore, the mechanical properties of the microparticles have not been characterized, and the interaction of the two phases under load has not yet been investigated. Due to the high cost of testing, finite element analysis has been widely used to investigate the local mechanical behavior of materials. Bartoli [16] proposed a method for estimating the local strain distribution in two phases of materials. The results show that the strain concentration exists in band form. Gu [18] analyzed the residual stress of an arc welded joint of a duplex stainless steel flat electrode via the finite element method and analyzed the residual stress distribution of different paths. However, most of the existing studies are not generalized, and do not analyze the distribution and evolution of local strains in the material.

In this paper, the local strain distribution and evolution characteristics of duplex stainless steel during the tensile process are discussed using the digital image correlation (DIC) technique. Uniaxial indentation tests were performed on the austenite and ferrite phases of duplex stainless steel using the nanoindentation test method, and the mechanical properties of the two phases were investigated. Then, based on the finite element inversion method, the nanoindentation process was simulated to characterize the stress–strain relationships and material parameters of the two phases. Finally, the local micro-deformation and stress distribution of the duplex stainless steel was simulated by the finite element method based on the material parameters from the finite element inversion method and the RVE obtained from the DIC experiments. The regularity of the local stress–strain behavior of the dual-phase steel and the interaction between grains and grain boundaries were further explored.

## 2. Experimental and Numerical Simulation Methods

### 2.1. Nanoindentation Experiments

Commercial duplex stainless steel produced by Baoshan Iron and Steel Co., Ltd., Shanghai, China was used as the study object. A 20 × 20 × 8 mm duplex stainless steel sample with clear grain boundaries was obtained by hand grinding, mechanical polishing, and slight electrolytic etching in a 20% NaOH solution. Then, the sample was observed under an optical microscope. The nanoindentation tests were conducted on a Nano Indenter G200 (Agilent Technologies, Santa Clara, CA, USA), with the XP Berkovich indenter selected. The experimental temperature was 25 °C. Six unidirectional indentation experiments were performed on austenite and ferrite in the region away from the grain boundaries using a displacement-controlled mode with a peak displacement of 1500 nm and a thermal drift of 0.05 nm/s.

### 2.2. DIC Experiments

**Figure 1:**
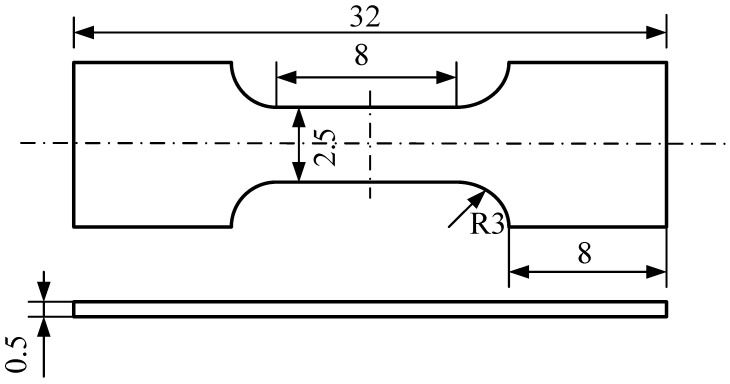
Schematic drawing of the specimen for tensile test.

**Figure 2:**
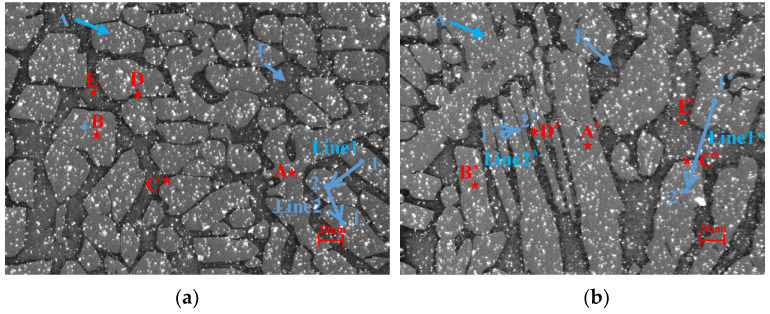
Representative areas after applying speckle: (**a**) uniform grain distribution area (1#) and (**b**) vertical fence-shaped grain area (2#).

In order to fully match the in situ stretching table, the samples processed in the manner of Section 2.1 were machined to the dimensions shown in Figure 1. At the same time, speckle was added to the working section to facilitate DIC observation. The experiments were performed on an in situ stretching table (Deben UK Ltd., Suffolk, UK). The sample was clamped symmetrically, loaded at a rate of 0.1 mm/min, and interrupted for photographs; the experimental temperature was 25 °C. After stretching to a certain load level, the load level remained unchanged for a while and the microstructure was photographed immediately with a scanning electron microscopy (SEM) (Zeiss, Oberkochen, Germany, Magnified 750 times). The regions with independent and uniformly distributed grains (1#, Figure 2a) and the regions with relatively narrow and roughly vertical grain-shaped grains (2#, Figure 2b) were selected as the study area and photographed by SEM for different sets of loading levels during the experiment. Finally, the strain evolution and local strain contour plot of the local region under each load level were calculated at each point using Vic-2D software with the corresponding difference method.

### 2.3. Determination of the Mechanical Parameters for the Two Phases

Before the local stress–strain behavior of duplex stainless steel can be simulated and described by finite element methods, it is crucial to obtain the mechanical properties of the austenite and ferrite phases. Dao [19] et al. used a power function strengthening model (shown in Equation (1)) to characterize the elastoplastic properties of metallic materials. The load-displacement derived from the indentation test combined with dimensional analysis and the finite element method can predict the yield strength and hardening index quite well, and has been widely developed and used. Furthermore, the inversion method proposed by Ma [20] can be used to compare the finite element simulation results and the experimental results with improved efficiency and accuracy.

(1) $$\left\{\begin{array}{c} \;\;\;\;\;\;\;E\varepsilon \;\;\;\;\;\;\;\;\;\left(\sigma \leq \sigma_{y}\right)\\ \sigma_{y}{\left(1+\frac{E}{\sigma_{y}}\varepsilon_{p}\right)}^{n}\;\;\;\;\;\;\left(\sigma >\sigma_{y}\right) \end{array}\right.$$

**Figure 3:**
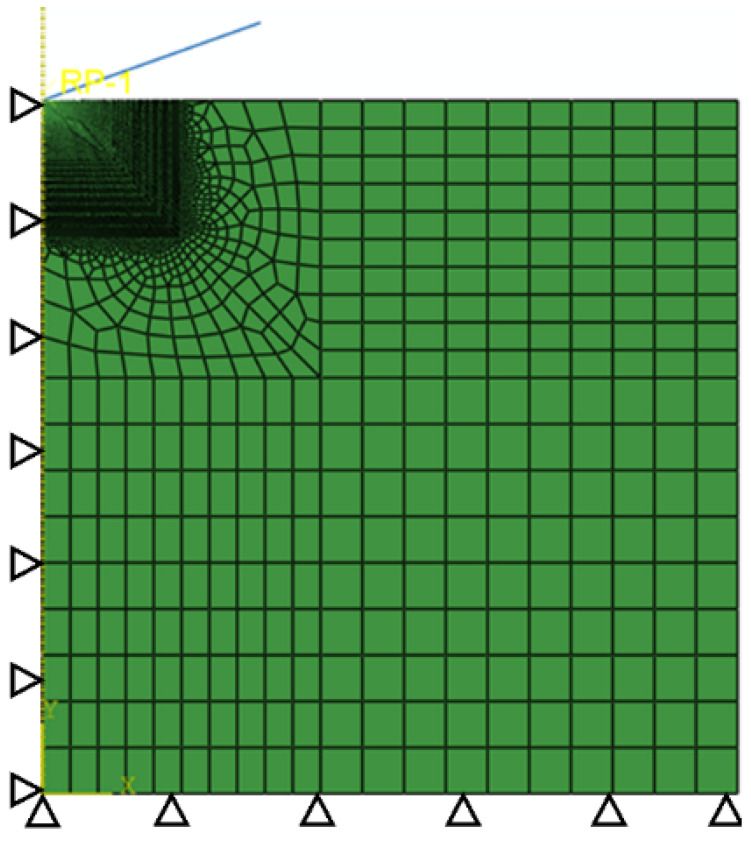
The boundary conditions and meshing of the finite element model.

Based on the nanoindentation experiment, the elastic modulus *E* and hardness *H* of both phases were obtained. Then, the unidirectional nanoindentation processes of the austenite phase and ferrite phase were simulated based on the finite element inversion method to determine the characteristic stress $\sigma_{r}$, characteristic strain $\varepsilon_{r}$, effective strain $\varepsilon_{p}$, yield stress $\sigma_{y}$, and hardening exponent n for both phases. It should be mentioned that in the finite element inversion method the indentation load–indentation depth curves obtained from the simulations and experiments were compared and analyzed for errors, through which the parameters were continuously updated by optimization iterations until the simulation results and experimental results met the error requirements. The established finite element axisymmetric model is shown in Figure 3. The Berkovich indenter [21] is equivalent to a conical indenter with a vertex angle of 140.6°, and the mesh is refined near the tip of the indenter. The element type was a CAX4, the constraint in the vertical direction is applied to the bottom of the model, the constraint in the horizontal direction is applied to the left symmetry axis, and displacement control is applied to the reference point. In addition, the displacement condition is applied on the reference point.

### 2.4. Finite Element Analysis of DIC Experiment

**Figure 4:**
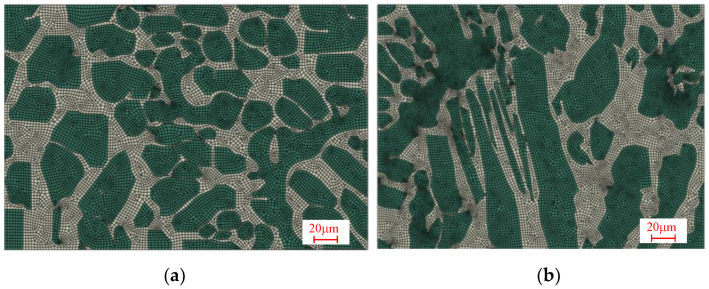
Geometric model and meshing of representative regions: (**a**) uniform area of grain ellipse (1#) and (**b**) vertically fenced area (2#).

In the DIC experimental observation, two representative regions were selected, one of which was the uniform distribution area with small grain size and the other the vertical fence-shaped grain area with large grain size, as shown in Figure 2. Based on the 1# and 2# regions captured by SEM(shown in Figure 2), the vectorization method was performed for the geometric modelling, then the finite element model was established using the finite element software, as shown in Figure 4.

**Figure 5:**
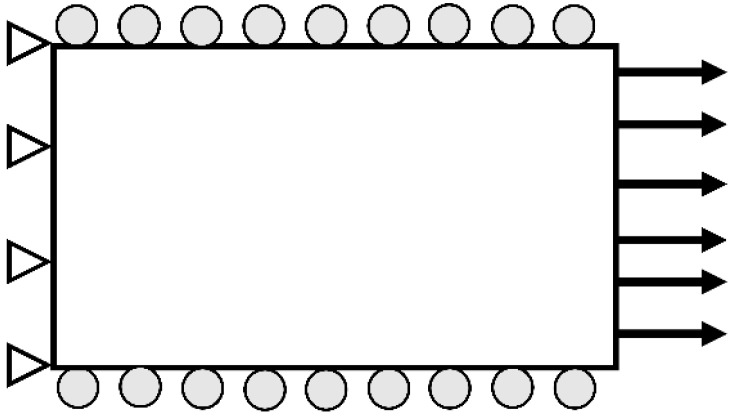
Schematic diagram of boundary conditions for the RVE.

Sheet specimens were used for the tensile test in this study. Because sheet specimens are relatively thin compared to their in-plane dimensions and are subjected to in-plane loading during uniaxial tensile tests, a specimen may generally be considered to be in a plane stress state. Therefore, two-dimensional plane stress CPS3 elements are adopted in this study to simulate the in-plane tensile behavior of the duplex stainless steel. The material properties of the austenite and ferrite phases are provided by the parameters and stress–strain curves obtained in Section 4.1. In the process of finite element simulation, the multilinear elastoplastic constitutive model was employed for stress–strain description of the austenite and ferrite phases. Simplified periodic boundary conditions were adopted for the RVE, as illustrated in Figure 5. All the nodes on the right side of the RVE were subjected to the same displacement in horizontal direction. Meanwhile, all the nodes on the left side were constrained in the horizontal direction. In addition, because the RVE is generated based on a sufficiently small microstructure level, all nodes on the top and bottom surfaces receive multi-point constraints, as shown in Figure 5, to ensure that they have the same displacement in the vertical direction. The above-mentioned treatment is consistent with that reported by Sun et al. [22].

## 3. DIC Experiment Results and Analysis

### 3.1. Strain Evolution Analysis

**Figure 6:**
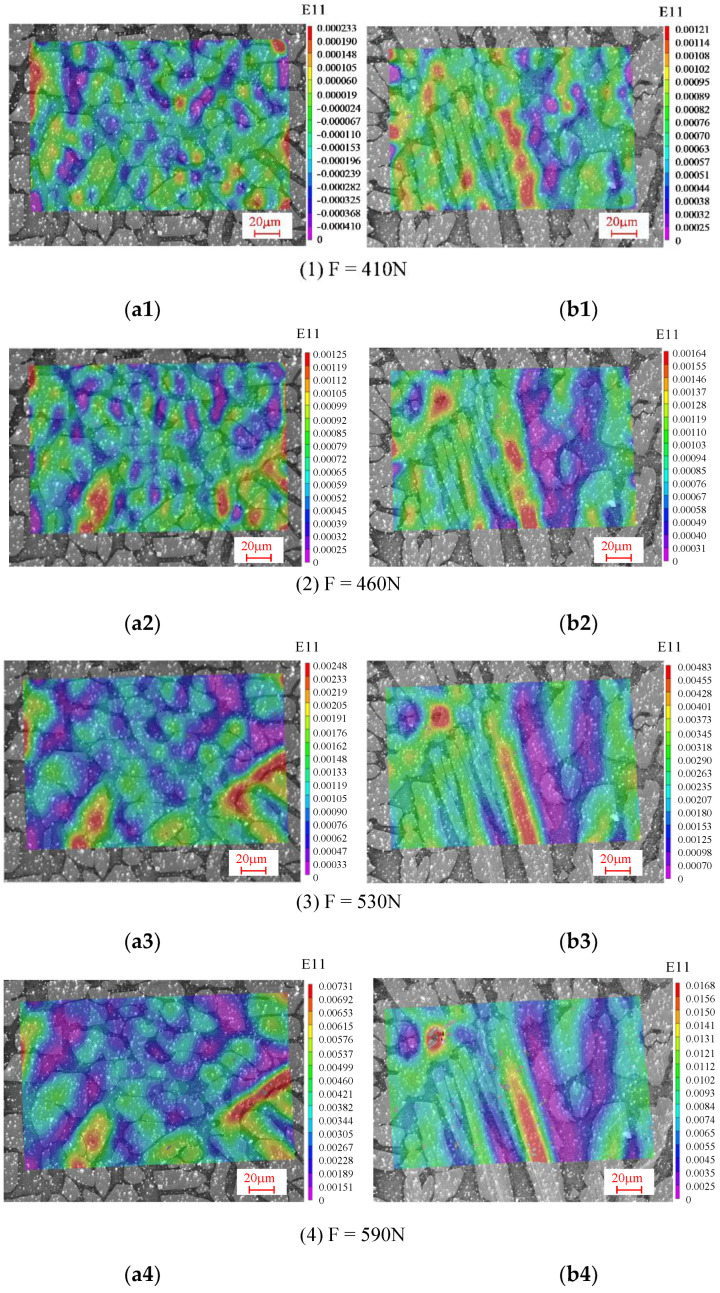

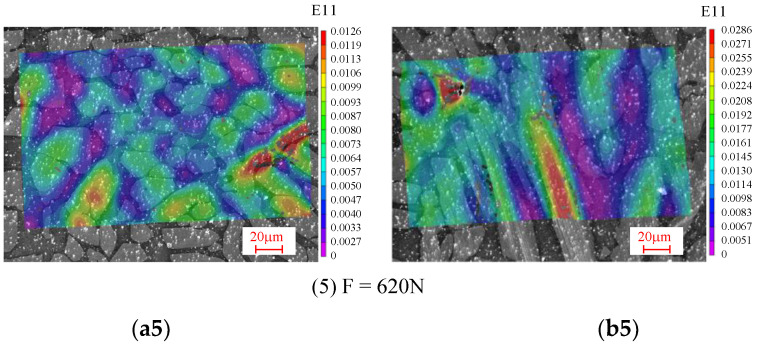
Schematic diagram of strain distribution in regions under different loads: (**a1**–**a5**) 1# and (**b1**–**b5**) 2# regions.

During the experiments, the speckle patterns were taken intermittently under the loads of 410N, 460N, 530N, 590N, and 620N. The strain distribution contour plots in the tensile direction (the horizontal direction in Figure 2) were obtained by Vic-2D software analysis, as shown in Figure 6. The 1# area is shown in Figure 6a, and the 2# area is shown in Figure 6b.

The engineering stress–strain curve can be calculated from the load–displacement curve recorded in the specimen uniaxial tensile test. The engineering strain can be obtained by dividing the effective length of the sample, and the engineering stress can be obtained by dividing the load by the effective cross-sectional area of the specimen. In fact, the calculated strain is larger than the true case, and the calculated elastic modulus is not within the normal range due to the deformation of fixture. Therefore, the true strain needs to be obtained by dividing the engineering strain by a correction value. The correction value is obtained by comparing the elastic stage of the load–tensile length curve with the conventional elastic modulus [23,24]:

(2) $$\varepsilon =\frac{\varepsilon_{T}}{f\left(s\right)}$$

where ε is the macroscopic strain,$\;\varepsilon_{T}$ is the strain value measured in the micro-sample, and $f\left(s\right)$ is the correction value; thus, the correction value is calculated as 7.5 [24].

The A grains in region 1# are elliptical and relatively evenly distributed. Although there is no significant change in the size and direction of the grains under in situ stretching, the strain in the tensile direction is concentrated in the A region due to the relatively softer property. The austenite grains in the 2# area are mostly fence-shaped, and the size and direction of the grains do not change significantly. The strain distribution in the tensile direction is concentrated on the austenite as well. The strain distribution is closely related to the distribution of the austenite and ferrite phases. The strain tends to be smaller in the area where the austenite distribution is sparse and the ferrite is concentrated, while in the region where the austenite phase is densely distributed the strain is larger. In addition, the distribution is closely related to the shape of the grains. Comparing the strain values of 1# and 2# under the same load condition in Figure 6, it can be seen that the strain in the austenite grain is relatively large and the peak strain of the 2# region is larger than that of the 1# region. Comparing the grains in areas 1# and 2#, the austenite grain boundary is more prone to strain concentration, with much more arris. In addition, the strain distribution is related to the load level. The difference between the strain in austenite and ferrite grains is small under low loading conditions, as shown in Figure 6(a1,b1,a2, b2). As the load level increases, the strain concentration becomes more obvious, and the strain becomes mostly concentrated on the austenite grains, as shown in Figure 6(a3–a5,b3–b5). Furthermore, as the load increases, the grains in the 1# and 2# regions are stretched, and the deformation rotates in the counter-clockwise direction.

### 3.2. Analysis of Local Deformation

In order to further investigate the local strain distribution characteristics of duplex stainless steel, five particular positions were selected to further investigate the strain evolution with increasing load conditions in combination with the shape and distribution of individual grains and strain distribution contour plots in the tensile direction. Figure 2a (Figure 2b) shows micrographs of the 1# (2#) region: austenite grains are mostly elliptical, point A and A* are inside the elongated austenite, point B and B*are inside the ordinary austenite, and point C and C* are in the austenite and ferrite junction boundary. Point D and D*are located inside the elongated ferrite, and point E and E* are located inside the ordinary ferrite.

**Figure 7:**
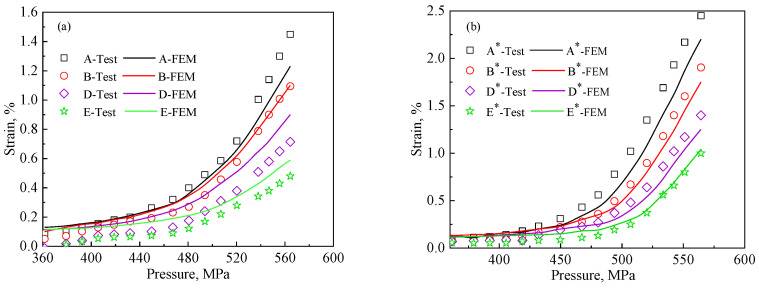
Strain evolution of special points with increasing load in the test: (**a**) region 1# (**b**) region 2#.

The strain–load curves of the selected points are shown in Figure 7. As can be seen in Figure 7, the strain magnitude in the tensile direction of all the points in the 1# region and 2# region increases with the loading level increasing. In addition, there is a similar change in phase austenite and phase ferrite in the elastic phase. However, as the load increases and enters the plastic phase, the strain is gradually concentrated in phase austenite. This is due to the relatively low modulus of elasticity of phase austenite. Therefore, the strain in austenite is slightly lower than that in ferrite at the elastic stage. Because austenite is softer than ferrite, the strain-strengthening exponent is lower than that of ferrite. In addition, the strain of phase austenite is larger than that of phase ferrite. After entering the plastic phase, the strain becomes more and more pronounced with the increase of load. This is consistent with the results of nanoindentation experiments and strain contour analysis in the tensile direction.

As shown in Figure 7, the strain value increases gradually from austenite to ferrite phase. Because austenite (D,E) is relatively soft, the strain in the austenite phase is always larger than that in the ferrite phase (A,B). At the junction boundary of austenite and ferrite (C), interaction exists between the austenite and ferrite grains. Therefore, the strain value is between that in austenite (A,B) and ferrite (C,D). In addition, the strain values are influenced by the grain shape. Narrow austenite grains (shown in Figure 6) are distributed in the austenite concentrated region, which tend to share larger strain. Thus, the strain value in narrow austenite grains (B and B*) is greater than that in normal austenite grains (A and A*). Similarly, the long and narrow ferrite grains are located in the austenite concentration region, which bear a larger strain. In addition, there is significant interaction between the boundaries of austenite and ferrite (C and C*), leading to larger strains than the general ferrite (D and D*), which is consistent with the results in Section 2.1. In general, the stress concentration of austenite and ferrite occurs on the narrow and long grains.

**Figure 8:**
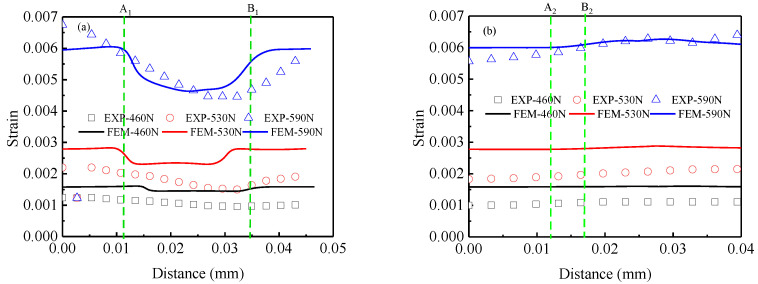

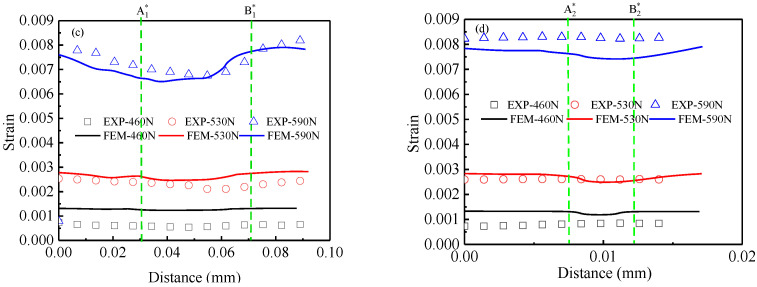
Strain distribution along different paths in the test: (**a**) path Line1 in the 1# region, (**b**) path Line2 in the 1# region, (**c**) path Line1* in the 2# region, (**d**) path Line2* in the 2# region.

In order to further investigate the influence of the grain distribution and the interaction between austenite and ferrite grains on the strain distribution in duplex stainless steel, two kinds of paths were studied according to the austenite grain spacing, as shown in Figure 8. One kind is the paths between two dispersed austenite grains (where the grain spacing is large), such as the blue lines 1 and line 1* in Figure 2a. The other kind is the paths between two compact austenite grains (where the grain spacing is small), such as the blue lines 2 and line 2* in Figure 2b. Figure 8a,b shows the strain distribution curves along the path direction of line 1 and line 2 from 1 (2) to 2 (1) points (shown in Figure 2a) at loading levels of 460 N, 530 N, and 590 N, respectively. Figure 8c,d shows the strain distribution curves along the path direction of line 1* and line 2* from 1* to 2* points (shown in Figure 2b) at loading levels of 460 N, 530 N, and 590 N, respectively. The A_1_ (A_2_, $A_{1}^{*}$, $A_{2}^{*}$) and B_1_ (B_2_, $B_{1}^{*}$, $B_{2}^{*}$) lines shown in Figure 8 denote the intersection points of the boundary of the austenite grain and the paths. It can be seen from Figure 8a,c that in both region 1# and region 2#, the strain magnitude along the paths tends to become smaller when passing into the ferrite grain from the interior of the austenite grain. Then, in the ferrite region, the strain magnitude evolves as a parabolic line before crossing into another austenite grain interior, during which the strain magnitude first decreases and then increases. This phenomenon is induced due to the strength difference between the austenite and ferrite grains. In addition, no obvious mutation of strain magnitude exists at the grain boundary, indicating that there is interaction between the austenite and ferrite grains that affects the local mechanical properties near the grain boundary. Furthermore, it can be observed in Figure 8b,d that when the A grain spacing is small, the two adjacent austenite grains in both the 1# region and 2# regions have a very high strain value close to that in the intermediate transition ferrite. This further indicates that interaction between the two phases that can affect each other’s local material properties, which is consistent with the results of analysis in Figure 8a,c.

## 4. Finite Element Analysis Results and Discussion

### 4.1. Material Parameters of the Austenite and Ferrite Phase

**Figure 9:**
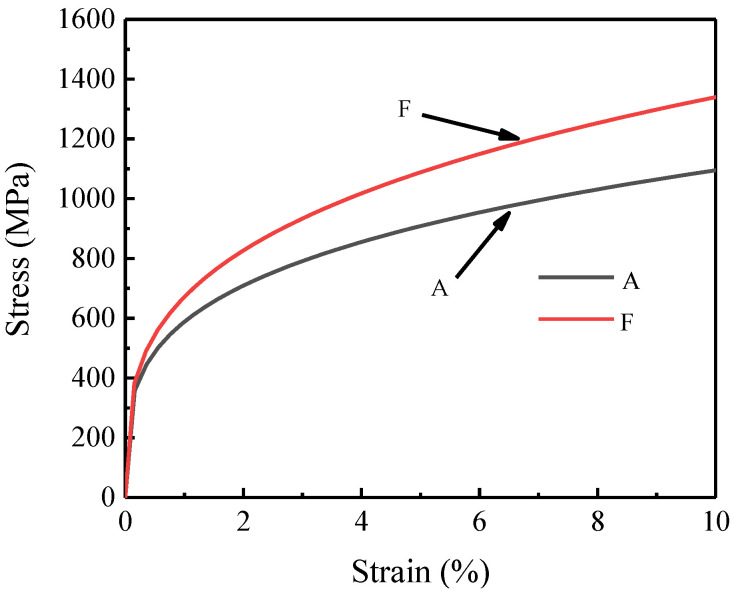
Tensile curves of austenite and ferrite phases.

**Figure 10:**
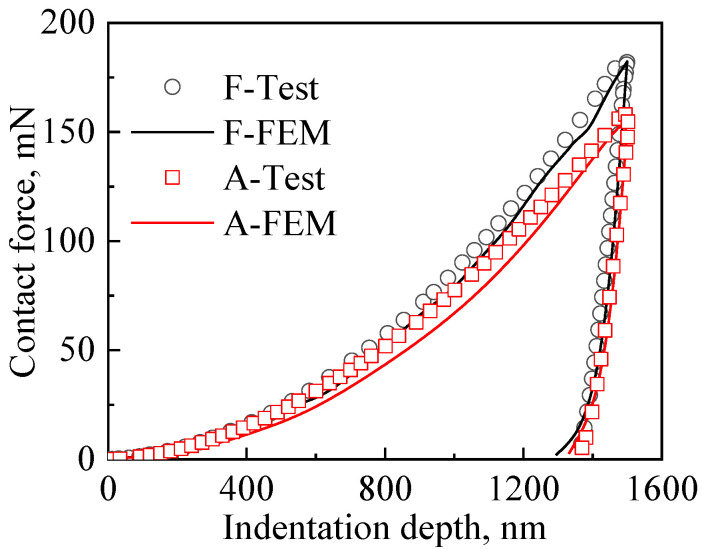
Load–pressing depth curve for nanoindentation experiments and finite element simulation results.

In this section, the average values of six sets of nanoindentation experimental data are employed for the finite element inversion method. The final determined strain strengthening index and yield strength for austenite and ferrite are 0.27, 358.2 GPa and 0.3, 381.2 GPa, respectively. The tensile stress–strain curves of the austenite and ferrite phases obtained by the finite element inversion method are shown in Figure 9. It can be seen from the figure that austenite enters the plastic stage earlier than ferrite, and austenite is softer than ferrite and generally bears more strain. It should be mentioned that the letters A and F in Figure 9 and Figure 10 denote the austenite and ferrite phases, respectively.

Finite element calculations of the nanoindentation process were performed for the austenite and ferrite phases to obtain the indentation depth–load curve. Figure 10 shows the comparison of the simulated and experimental average results for the six groups. It can be seen in Figure 10 that the load–indentation depth curve obtained from the finite element numerical simulations fits well with the experimental results, indicating that the constitutive relationship for the austenite and ferrite phases calculated by the finite element inversion method is correct. According to the experimental and simulation results, the maximum load of ferrite phase is greater than the A phase at the same indentation depth, indicating that austenite is softer and deforms more easily than the ferrite phase.

**Figure 11:**
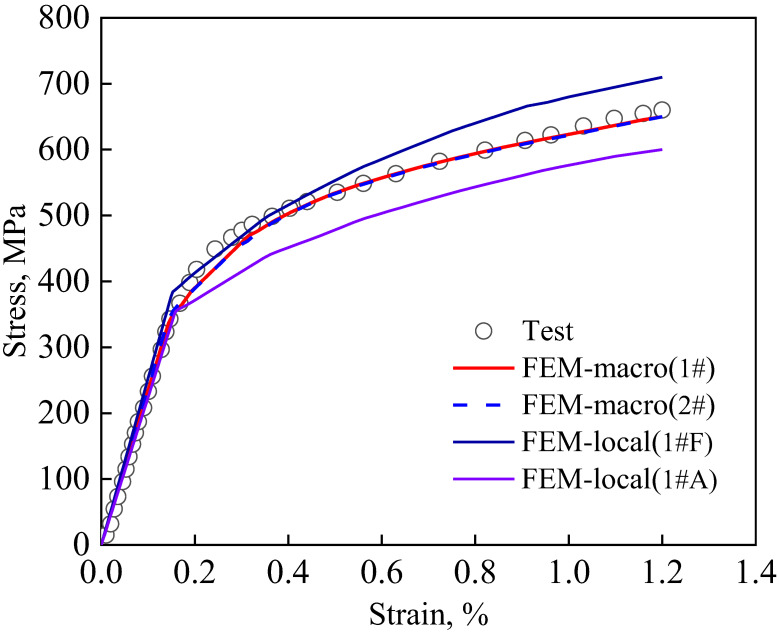
Comparison of tensile curves between experimental and simulation results.

The macro stress–strain curves obtained from the finite element simulation method introduced in Section 2.4 for the representative regions 1# and 2# are shown in Figure 11. The experimental results from the DIC test are also shown in Figure 11. It can be seen that good agreement between the simulation and experimental results is achieved using either representative region 1# or 2#. In addition, Figure 11 shows the local stress–strain curves for the highest stress points in the austenitic and ferrite phases during uniaxial stretching. It can be seen that the stress in the austenitic phase is lower than the macroscopic stress in the duplex stainless steel at the same macroscopic stress–strain level, while the stress in the ferritic phase is higher than the macroscopic stress, reflecting the strengthening effect of the ferritic phase on the duplex stainless steel.

### 4.2. Micro-Plastic Deformation Behavior and Evolution Analysis

**Figure 12:**
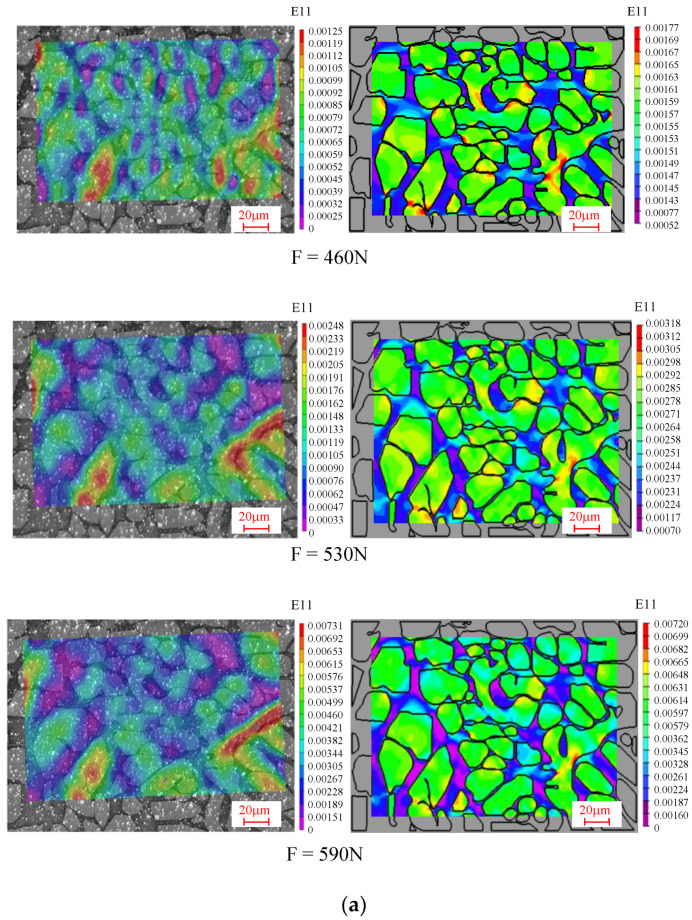

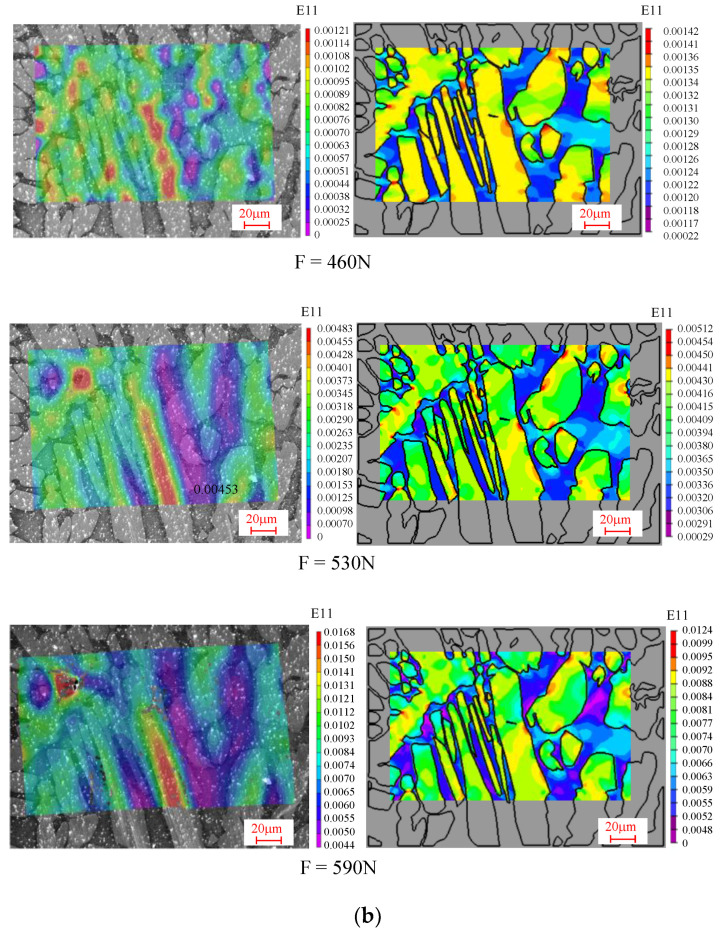
Comparison of the strain distribution contours of FEA results and experimental results under different loads: (**a**) 1# region, (**b**) 2# region.

The strain distributions in the tensile direction of the 1# and 2# regions were calculated by the finite element method under different loading levels (410N, 460N, 530N, 590N, and 620N), and are compared with the experimental results in Figure 12a,b. It can be seen that the strain contour plots obtained by experiment are consistent with those obtained from the finite element simulations. The mechanical characteristics of the duplex stainless steel analyzed by the experimental strain contour plot are apparent in the finite element simulation results as well. Thus, the finite element analysis method adopted in this paper is feasible, and the microstructure distribution can be used to predict the strain distribution of the material under macroscopic loading. Simultaneously, Figure 7 shows the simulated strain evolution curves of the A-E characteristic points with increasing loading, which is consistent with the experimentally derived pattern. Moreover, the finite element method can compensate for the limitation of the DIC technique, which is highly versatile and can be used to predict the mechanical properties for a variety of materials. The finite element method can help researchers to discuss the local stress distribution in different regions.

### 4.3. Analysis of Microscopic Stress Distribution

**Figure 13:**
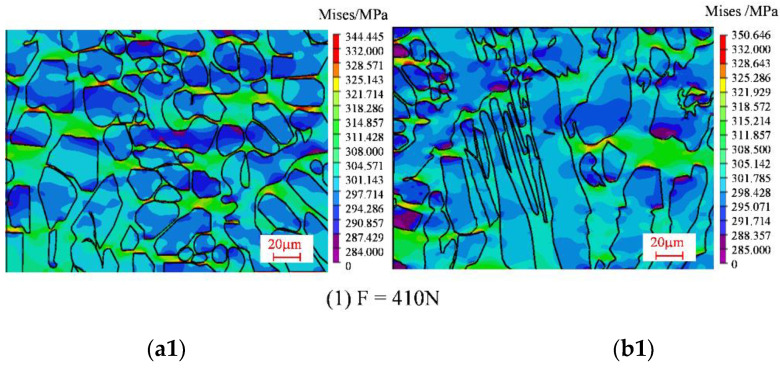

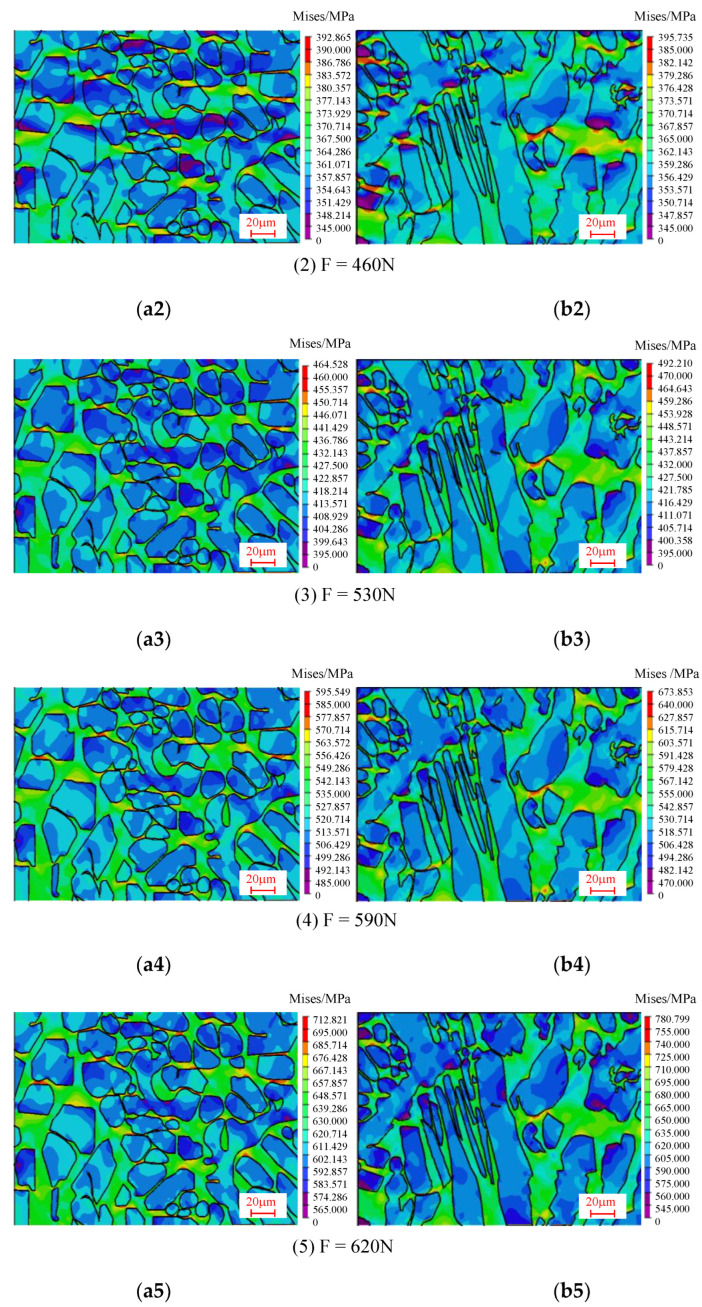
Stress distribution contours under different loads: (**a1**–**a5**) 1# region, (**b1**–**b5**) 2# region.

**Figure 14:**
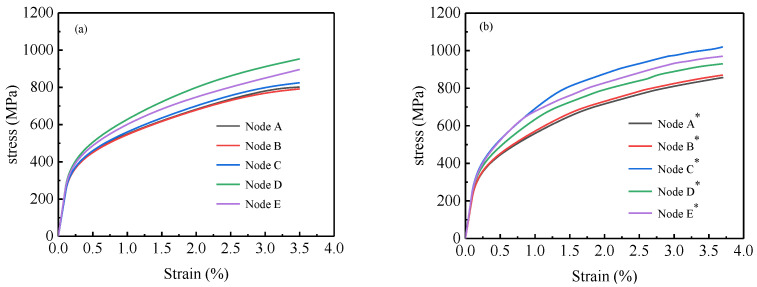
Stress–strain curves at the special points in numerical simulation: (**a**) 1# region, (**b**) 2# region.

The stress distribution diagrams for the 1# and 2# regions obtained by the finite element simulations under loading levels of 410N, 460N, 530N, 590N, and 620N are shown in Figure 13. The stress–strain curves for the characteristic points A–E (A*–E*) in Figure 2 are shown in Figure 14.

According to the stress distribution map, the larger stress is concentrated on the ferrite phase in both region 1# and region 2# under a certain load, indicating that the strength of the ferrite is relatively higher. Pronounced growth of stress appears as the loading level increases. It can be concluded from Figure 13 that the internal stress in ferrite phases is smaller than that around the grain boundaries, and the stress in the austenite phase is larger than that around the grain boundaries. Obvious stress concentration occurs at higher load levels due to the interaction between the austenite and ferrite phases. Furthermore, the ferrite grains close to the austenite grains tend to bear higher loadings, which is related to higher stress concentration. The stress concentration regions are distributed in strip shapes and extend along the stretching direction. The stress concentrations in the ferrite phase occur in the regions with high austenite grain density, and appear much more obvious as the loading increases. A notable feature can be identified in that the stress concentration value of the 2# region is larger than that in the 1# region due to the different grain shapes. Compared with the grains in the 1# region, the austenite grains in the 2# region are fence-shaped and the grain surface is sharper. Furthermore, stress concentrations are more obvious as the loading level increases.

Figure 14a shows the stress–strain curves for points A, B, C, D, and E in the 1# region, while Figure 14b shows the stress–strain curves for points A*, B*, C*, D*, and E* in the 2# region. The 1# region and the 2# region exhibit the same characteristics: the curves from high to low are D, E, C, A, and B, which is consistent with the experimental analysis mentioned earlier.

## 5. Conclusions

In this paper, the local strain and stress distribution and evolution characteristics of duplex stainless steel during the tensile process were studied based on the DIC technique and the finite element method. Meanwhile, a finite element inversion method combined with a nanoindentation approach was developed to obtain the material parameters of the ferrite and austenite phases. The simulation results from the developed numerical methods fit well with experimental observations from the DIC technique. The results show that the high strain zone is mainly located in the austenite phase; however, the high stress zone is mainly located in the ferrite grains and the grain boundaries. There is a strong interaction between the austenite and ferrite phases, and the interaction between the two phases affects the local material properties of each phase near the grain boundaries. This study effectively reveals the two-phase interaction and local failure mechanism of duplex stainless steel, and may provide a reference for material preparation and safety design of related structures.

## Data Availability

Not applicable.
